# The Infrared Thermography Toolbox: An Open-access Semi-automated Segmentation Tool for Extracting Skin Temperatures in the Thoracic Region including Supraclavicular Brown Adipose Tissue

**DOI:** 10.1007/s10916-022-01871-7

**Published:** 2022-11-02

**Authors:** Aashley S. D. Sardjoe Mishre, Maaike E. Straat, Borja Martinez-Tellez, Andrea Mendez Gutierrez, Sander Kooijman, Mariëtte R. Boon, Oleh Dzyubachyk, Andrew Webb, Patrick C. N. Rensen, Hermien E. Kan

**Affiliations:** 1 Department of Medicine, Division of Endocrinology, Leiden University Medical Center, Leiden, the Netherlands; 2 Department of Radiology, C.J. Gorter Center for High Field MRI, Leiden University Medical Center, Leiden, the Netherlands; 3 Department of Radiology, Division of Image Processing (LKEB), Leiden University Medical Center, Leiden, The Netherlands; 4 Department of Cell and Chemical Biology, Section Electron Microscopy, Leiden University Medical Center, Leiden, The Netherlands; 5 Department of Education, Faculty of Education Sciences and SPORT Research Group (CTS-1024), CERNEP Research Center, University of Almería, Almería, Spain; 6 Department of Biochemistry and Molecular Biology II, “José Mataix Verdú” Institute of Nutrition and Food Technology (INYTA), Biomedical Research Centre (CIBM), University of Granada, 18016 Granada, Spain; 7 CIBER Fisiopatología de la Obesidad y la Nutrición (CIBEROBN), Biohealth Research Institute in Granada (Ibs. GRANADA), 28029 Madrid, Spain

**Keywords:** Infrared thermography, Non-rigid image registration, Semi-automated analysis, BAT

## Abstract

Infrared thermography (IRT) is widely used to assess skin temperature in response to physiological changes. Yet, it remains challenging to standardize skin temperature measurements over repeated datasets. We developed an open-access semi-automated segmentation tool (the IRT-toolbox) for measuring skin temperatures in the thoracic area to estimate supraclavicular brown adipose tissue (scBAT) activity, and compared it to manual segmentations. The IRT-toolbox, designed in Python, consisted of image pre-alignment and non-rigid image registration. The toolbox was tested using datasets of 10 individuals (BMI = 22.1 ± 2.1 kg/m^2^, age = 22.0 ± 3.7 years) who underwent two cooling procedures, yielding four images per individual. Regions of interest (ROIs) were delineated by two raters in the scBAT and deltoid areas on baseline images. The toolbox enabled direct transfer of baseline ROIs to the registered follow-up images. For comparison, both raters also manually drew ROIs in all follow-up images. Spatial ROI overlap between methods and raters was determined using the Dice coefficient. Mean bias and 95% limits of agreement in mean skin temperature between methods and raters were assessed using Bland–Altman analyses. ROI delineation time was four times faster with the IRT-toolbox (01:04 min) than with manual delineations (04:12 min). In both anatomical areas, there was a large variability in ROI placement between methods. Yet, relatively small skin temperature differences were found between methods (scBAT: 0.10 °C, 95%LoA[-0.13 to 0.33 °C] and deltoid: 0.05 °C, 95%LoA[-0.46 to 0.55 °C]). The variability in skin temperature between raters was comparable between methods. The IRT-toolbox enables faster ROI delineations, while maintaining inter-user reliability compared to manual delineations. (*Trial registration number (ClinicalTrials.gov)*: NCT04406922, [May 29, 2020]).

**Supplementary Information:**

The online version contains supplementary material available at 10.1007/s10916-022-01871-7.

## Introduction

Infrared thermography (IRT) is a non-invasive, safe and inexpensive imaging technique for assessing surface temperature. The working principle behind IRT is that all objects emit infrared radiation [1]. The intensity and wavelength of the emanated radiation can be used to calculate surface temperatures, which are displayed as colored heatmaps.

IRT is used to study the relation between thermal physiology and skin temperature in humans [2], and has been utilized for the diagnosis of breast cancer, diabetic neuropathy and peripheral vascular disorders [3]. There has been increasing interest in utilizing IRT for assessing thermogenic activity induced by brown adipose tissue (BAT) [4]. BAT is a thermogenic tissue found in mammals, with cold exposure being its most potent physiological activator [5, 6]. Upon activation, BAT combusts triglyceride-derived fatty acids and glucose, producing heat due to the presence of uncoupling protein 1 (UCP-1) in its mitochondria. Previous studies have employed IRT to assess BAT activity by measuring skin temperature in the supraclavicular region, the location of the largest BAT depot in humans [7–9].

Although IRT has been used for many clinical applications, it is challenging to standardize repeated measurements from a region of interest (ROI). Manual ROI delineations are time-consuming and have a poor spatial reproducibility [4, 10]. Fully automated ROI extraction methods have been developed for several anatomical regions [3, 10]. However, these methods rely on approaches such as clustering, thresholding or edge-detection, which cannot be easily applied to regions with irregular structures or low tissue contrast such as in the supraclavicular area [10]. Semi-automated ROI extraction methods, requiring some manual input, are faster than manual methods [11–13]. Law et al. developed a semi-automated ROI method for extracting supraclavicular skin temperature [11], which improved analysis speed while maintaining reproducibility of manual delineations.

However, these semi-automated ROI methods still require manual input for defining the ROI on all images within a dataset. This makes the analysis in studies with large cohorts and/or multiple interventions challenging, particularly when there are differences in subject orientation and positioning with respect to the thermal camera. These challenges can potentially be overcome by using non-rigid image registration, which enables a pixel-by-pixel overlap between the baseline image and every follow-up image. A single baseline ROI can be chosen, which can be directly transferred to all registered follow-up images. In this work, we have developed an open access semi-automated toolbox using non-rigid image registration for measuring skin temperatures in two regions of the thoracic area. We compared the toolbox with manual delineations for analysis time, ROI placement and inter-user reliability.

## Materials and Methods

### The Main Features of the IRT-Toolbox

The IRT-toolbox was implemented using Python (Python Software Foundation. Python Language Reference (v3.8.5). Thermal images were initially saved in JPEG format and subsequently converted to temperature maps using the Python package: Flir Image Extractor (v1.4.0). The ExifTool application was used to extract metadata from the thermal images [14]. We did not use any commercially available software development kit to analyse our images. The main features of the toolbox are: image pre-alignment, non-rigid image registration and semi-automated ROI segmentation.

### Image Pre-alignment and Non-rigid Image Registration

**Fig. 1:**
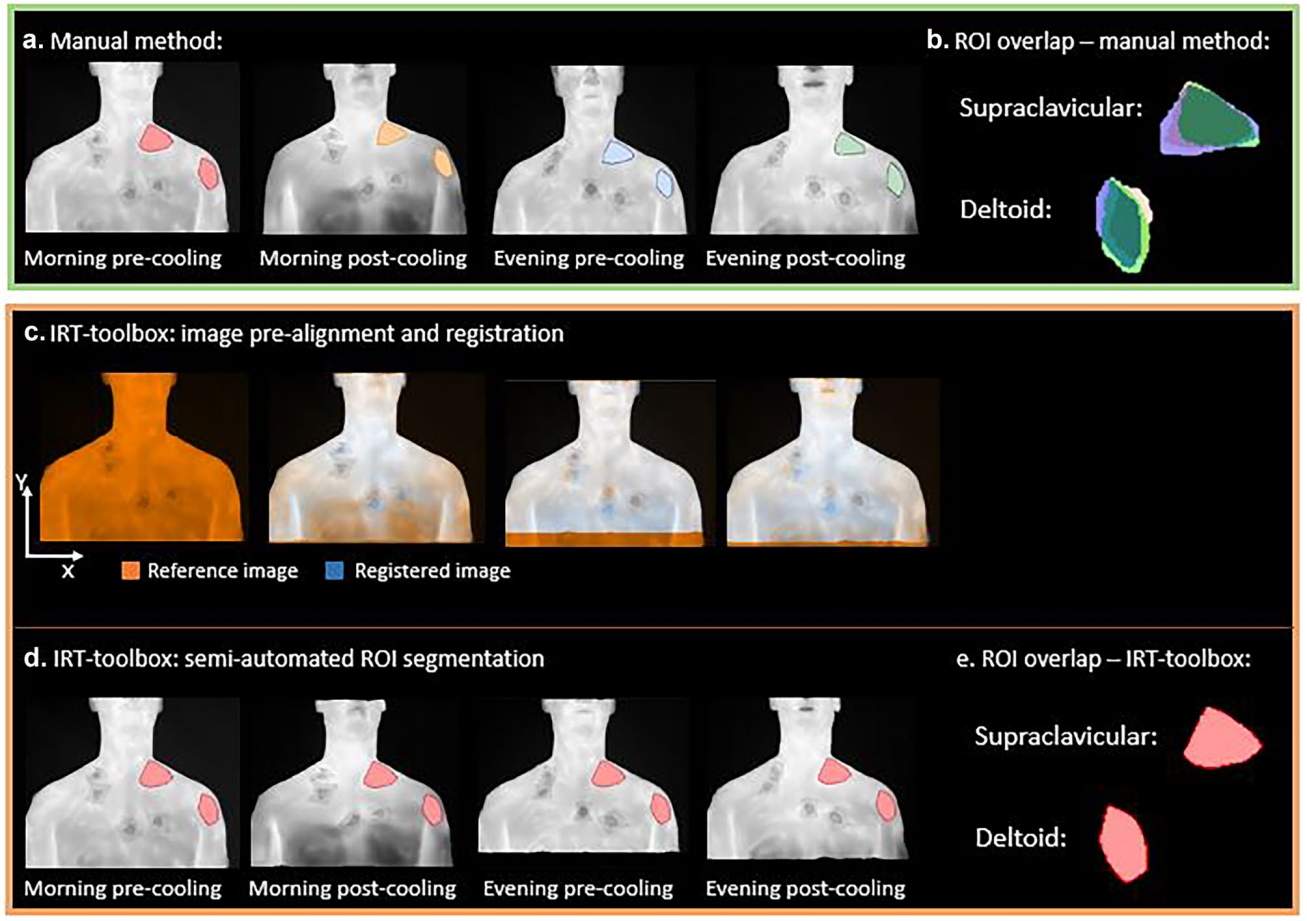
A schematic overview of manual delineations and the IRT-toolbox **a.** Manual method: regions of interest were manually delineated on the reference image (morning pre-cooling) and the follow-up images (morning post-cooling, evening pre-cooling and evening post-cooling) in each dataset. **b.** The overlap between manually-delineated ROIs in the supraclavicular and deltoid areas is shown for one participant. Follow-up ROIs were aligned with the reference ROI. **c.** IRT-toolbox: image pre-alignment and image registration were applied to obtain a pixel-wise image correspondence between registered follow-up images (blue) and the reference image (orange) within a single dataset. **d.** The ROI that was manually drawn on the reference image (step **a**), was mapped to the registered follow-up images. **e.** Mapping of the reference ROI removed the variability in manual ROI placement. ROI: region of interest

The challenge of repeated measurements is summarized in Fig. 1a and b. Four images were acquired at different times, and ROIs were manually drawn in the supraclavicular and deltoid regions on each image. The data show that there are differences in the position and orientation of the subject. These lead, as shown in Fig. 1b, to spatial differences in the ROIs drawn for the two areas in the four images. Figures 1c-e show the main features of the toolbox. In Fig. 1c, image pre-alignment was used to correct for large displacements between images: the neck was used as an anatomical landmark. The spatial coordinates of the neck were calculated for all images in each dataset and used to align each follow-up image to the baseline image. The neck coordinates were determined based on image thresholding, wherein the background was separated from the subject. Along each row of the image (*x*-direction; see Fig. 1), temperature differences were determined for consecutive pixels. This yielded a temperature gradient for each row, with minor differences in homogenous regions, and large peaks at transitions between the background (room temperature ~ 22 °C) and the body 34.3 ± 0.5 °C, at thermoneutrality and 29.9 ± 1.7 °C after cooling [15]. Pixels that were located between the transition peaks were given a value of 1 if their temperature values were above 25 °C (foreground pixels), whereas the other pixels in that same row were given a value of zero (background pixels). This was applied to all rows, until the body was fully separated from the background. Subsequently, the neck was located as the row corresponding to the smallest number of foreground pixels. The outer left *x* and *y* coordinates of the neck were used to shift each follow-up image towards the outer left *x* and *y* coordinates of the neck in the baseline image. The number of pixels were converted to centimeters, and reported as the amount of subject displacement prior to the image pre-alignment and registration steps.

The next step was to account for any geometric differences in the acquired images. Non-rigid image registration was integrated into the toolbox using the open-source image registration toolbox Elastix [16]. Non-rigid image registration enables a stepwise deformation of an image until it fully overlaps with a given reference image (*e.g.,* a baseline image). Registration parameters including the number of resolutions, number of iterations and the maximum step length of the optimizer were systematically optimized based on an image similarity metric, Mattes mutual information. As a quantitative indicator for the registration quality, it was evaluated whether the Mattes mutual information stabilized along the stepwise image deformation for each registration case. Image pre-alignment and non-rigid image registration were both implemented to obtain a pixel-wise image overlap between the reference image and every subsequent image within a participant’s dataset (Fig. 1c). As a result, ROIs only needed to be delineated on the reference image and this reference ROI could be subsequently transferred to the registered follow-up images (Fig. 1d), requiring no redrawing as shown in Fig. 1e.

### Study Subjects and Experimental Procedures

Data were obtained from four healthy males and six healthy females (BMI = 22.1 ± 2.1 kg/m^2^, age = 22.0 ± 3.7 years) who were enrolled in a clinical trial studying the circadian rhythm of cold-induced thermogenesis [15]. This study was approved by the Medical Ethical Committee of the Leiden University Medical Center and performed in accordance with the principles of the revised Declaration of Helsinki. Informed consent was obtained from all participants. The clinical trial was registered at ClinicalTrials.gov NCT04406922. The study design is extensively described elsewhere [15].

Participants underwent a personalized cooling procedure on two separate study days: in the morning (07:30 AM) and in the evening (07:30 PM). The examination room had an average room temperature of 22.0 ± 0.3 °C in the morning and 22.4 ± 0.4 °C in the evening. At the start of each study visit, an IRT image of the upper thorax/neck region was obtained using the FLIR T530 camera (FLIR Systems, Inc., Wilsonville, OR, USA). The camera was equipped with a 24° AutoCal^TM^ lens and had an image capture rate of 30 Hz. The distance between the camera and the participant was 1.5 meters and the camera was held orthogonally (90°) with respect to the participant. At the end of the cooling procedure, a second IRT image of the upper thorax/neck region was made. Overall, four IRT images were acquired per participant: morning pre-cooling, morning post-cooling, evening pre-cooling and evening post-cooling. The camera acquired an image with the size of 320 pixels (*x* direction) and 240 pixels (*y* direction) (Fig. 1), which was converted to centimeters [18] using the focal length (17 mm) and distance (1.5 m) between the camera and subject: the image size was 63 × 47 cm.

### ROI Segmentation

Two researchers (ASM and AMG; hereinafter ‘raters’) delineated ROIs in the supraclavicular and deltoid areas manually on the baseline image (morning pre-cooling, Fig. 1a). The supraclavicular depot was segmented using a triangular shape placed between the end of the neck and above the clavicular bone, which has been used in previous IRT studies to simplify manual delineations between raters [4]. The deltoid area was delineated by placing a polygon in the upper arm. The raters drew ROIs on all follow-up images to compare results with semi-automated segmentations. The manual morning pre-cooling ROI was used in the semi-automated analysis (IRT-toolbox), and directly applied to the registered follow-up images (*i.e.* morning post-cooling, evening pre-cooling and evening post-cooling) in each dataset. We refer to the morning pre-cooling image as the “reference image”, and the morning pre-cooling ROI as the “reference ROI”. ROIs were delineated in Matlab (version 2016a) using a custom-built function that enabled the user to draw polygons by mouse-clicking. ROIs were subsequently exported to Python for analysis. Of note, the current version of the IRT-toolbox is fully designed in Python, including the delineation step.

### Statistical Analysis

Mean and maximum skin temperatures were determined for all four imaging conditions from manually drawn ROIs and from the reference ROI directly applied to the registered images in each dataset (IRT-toolbox). These outcome measures were used to assess the variability between segmentation methods (intra-rater variability) and between raters (inter-rater variability). Data normality was tested using the Shapiro–Wilk test.

### The Assessment of Spatial Agreement and Temperature Outcomes Between the IRT-toolbox and Manual Segmentations

In the first analysis, we assessed the spatial overlap between methods, where we compared the overlap of the reference ROI (*i.e.* the ROI drawn on the reference image, that was directly used on registered follow-up images with the IRT-toolbox) with each manually drawn follow-up ROI. Since there were large displacements between the reference image and follow-up images, follow-up ROIs needed to be registered first to match the location of the reference ROI. The spatial overlap was then quantified using the Dice coefficient. The Dice coefficient was determined based on formula (1), wherein the overlapping area of two ROIs A and B is divided by the total number of pixels in both ROIs.

1 $$Dice(A,B)= \frac{2\left|A\cap B\right|}{\left|A\right|+\left|B\right| }=\frac{2\;*\;Area\; of\; overlap}{total\; number\; of\; pixels\; in\; both\; ROIs}$$

This overlap coefficient is reported for each anatomical region as mean and range: [min, max], wherein ROIs from both raters were included. The qualitative scores for the Dice coefficient were defined as 0–0.49 (poor), 0.5–0.69 (moderate), 0.7–0.89 (good), and > 0.9 (excellent) [20]. Subsequently, mean and maximum skin temperatures from the two raters were averaged to determine the mean skin temperature difference (mean bias) between methods, and to detect the variability between methods using the 95% limits of agreement (95%LoA) using a repeated measures Bland–Altman analysis (R Core Team v4.1.0 (2021), R Foundation for Statistical Computing, Vienna, Austria; R package: SimplyAgree).

### The Assessment of the Inter-user Reliability with the IRT-Toolbox Versus Manual Segmentations

In the second analysis, the performance of the IRT-toolbox and the manual method were assessed by evaluating the outcomes between raters. The Dice coefficient was used to determine the spatial agreement between ROIs delineated by the two raters. For the IRT-toolbox, the spatial agreement between the reference ROIs was assessed, resulting in 10 ROIs (*i.e*., 1 ROI per subject) being compared between raters. For the manual method, all manually drawn reference and follow-up ROIs were included, and therefore 40 ROIs (*i.e.,* 4 ROIs per subject) were compared between raters. Subsequently, the mean bias and 95% LoA in mean and maximum skin temperature between raters were determined for each method. Finally, the ROI drawing time of the supraclavicular region of the whole dataset was recorded for both segmentation methods for one rater. The registration time for a single image and the time required to register the entire dataset were also recorded. Statistical analyses were performed using SPSS (Statistical Package for the Social Sciences; v25).

## Results

### The IRT-Toolbox Reduces the ROI Delineation Time to a Single Image per Dataset

The average amount of subject displacement in the follow-up images relative to the reference image, prior to applying the IRT-toolbox, was 3.6 ± 2.5 cm along the *y* direction and 7.5 ± 5.4 cm along the *x* direction. After performing the image pre-alignment step, this initial displacement between images was reduced to zero. The optimized image registration parameters were: two-dimensional B-spline transform with a 10 × 10 mm^2^ grid, adaptive stochastic gradient descent with four resolutions, maximum step length of 0.5 and 450 iterations. In all datasets, image overlap and convergence of the image similarity index were visually assessed by one rater. Image registration took 38 s per image pair, and 21:13 min for the entire dataset. The total time for drawing supraclavicular ROIs for the entire dataset, *i.e.,* on all reference-and follow-up images, was 04:12 min using the manual method and 01:04 min with the IRT-toolbox.

### Intra-rater Analysis: The Agreement Between ROIs From the IRT-toolbox and Manual Method Showed a Wide Range, But Skin Temperature Differences Were Less Than 1 °C

First, we assessed the spatial overlap and skin temperature outcomes between the IRT-toolbox and manual segmentations. To assess the spatial overlap between methods, we compared the overlap between the reference ROI with each manually drawn follow-up ROI. We found a good agreement, albeit with a wide range, between reference ROIs and manually drawn follow-up ROIs in the supraclavicular area (Dice = 0.75, range: [0.42–0.93]), and a moderate agreement in the deltoid area (Dice = 0.66, range: [0.30–0.92]; see Table 1.

**Table 1:** The agreement in ROI placement between methods and raters 1. Methods: the spatial agreement between ROIs from the IRT-toolbox (reference ROI) and all manually-drawn follow-up ROIs. 2. Raters: the spatial agreement in ROI placement between raters when using the IRT-toolbox and the manual method. The spatial agreement was quantified with the Dice coefficient and the qualitative scores were: 0–0.49 (poor), 0.5–0.69 (moderate), 0.7–0.89 (good) and > 0.9 (excellent). Dice coefficient is presented as mean, range: [min, max]

	**1. Methods**	**2. Raters**	
		IRT-toolbox	Manual method
**Supraclavicular area**	Dice = 0.75, range: [0.42,0.93]	Dice = 0.73, range: [0.62–0.84]	Dice = 0.70, range: [0.56–0.86]
**Deltoid area**	Dice = 0.66, range: [0.30,0.92]	Dice = 0.75, range: [0.58–0.83]	Dice = 0.65, range: [0.38–0.83]

**Fig. 2:**
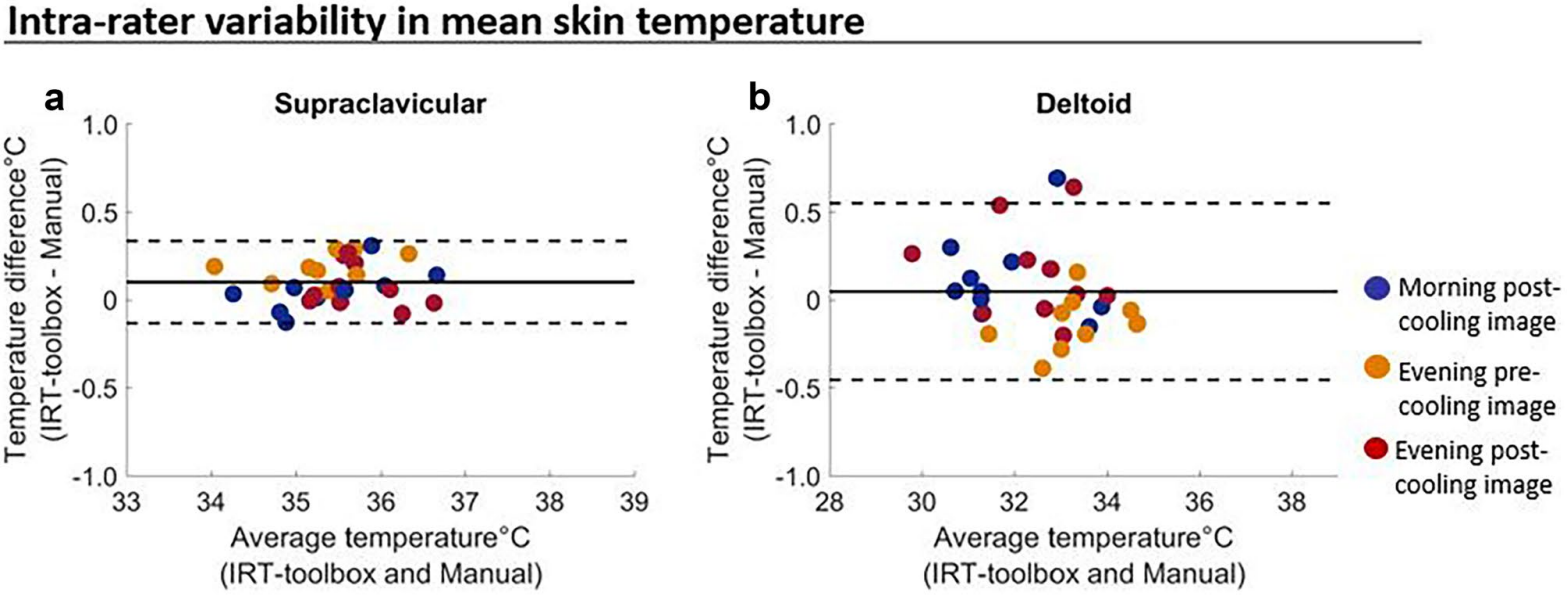
The mean difference and variability in mean skin temperature between methods. The mean difference and variability in mean skin temperature between methods for the supraclavicular area **a** and the deltoid area **b** are shown. Data for all ten participants are shown; different colors represent different imaging sessions. The solid line represents the mean difference and the dashed lines represent the upper and the lower 95% limits of agreement

A mean temperature difference of 0.10 °C, 95% LoA = [-0.13 °C,0.33 °C] was found for the supraclavicular area, and 0.05 °C, 95% LoA = [-0.46 °C,0.55 °C] for the deltoid area; see Fig. 2a, b. The results for maximum skin temperature are shown in Online resource 1; Fig. S1.

### Inter-rater Analysis: The IRT-Toolbox has a Similar Inter-user Variability to Manual Segmentations

Secondly, we assessed spatial agreement and temperature outcomes between the two raters for both methods. The IRT-toolbox revealed a good spatial overlap between reference ROIs from the two raters in the supraclavicular region (Dice = 0.73, range: [0.62–0.84]), and in the deltoid region (Dice = 0.75, range: [0.58–0.83]). For the manual method, a good spatial overlap in the supraclavicular region (Dice = 0.70, range: [0.56–0.86]), and a moderate overlap in the deltoid region (Dice = 0.65, range: [0.38–0.83]) were found; see Table 1.

**Fig. 3:**
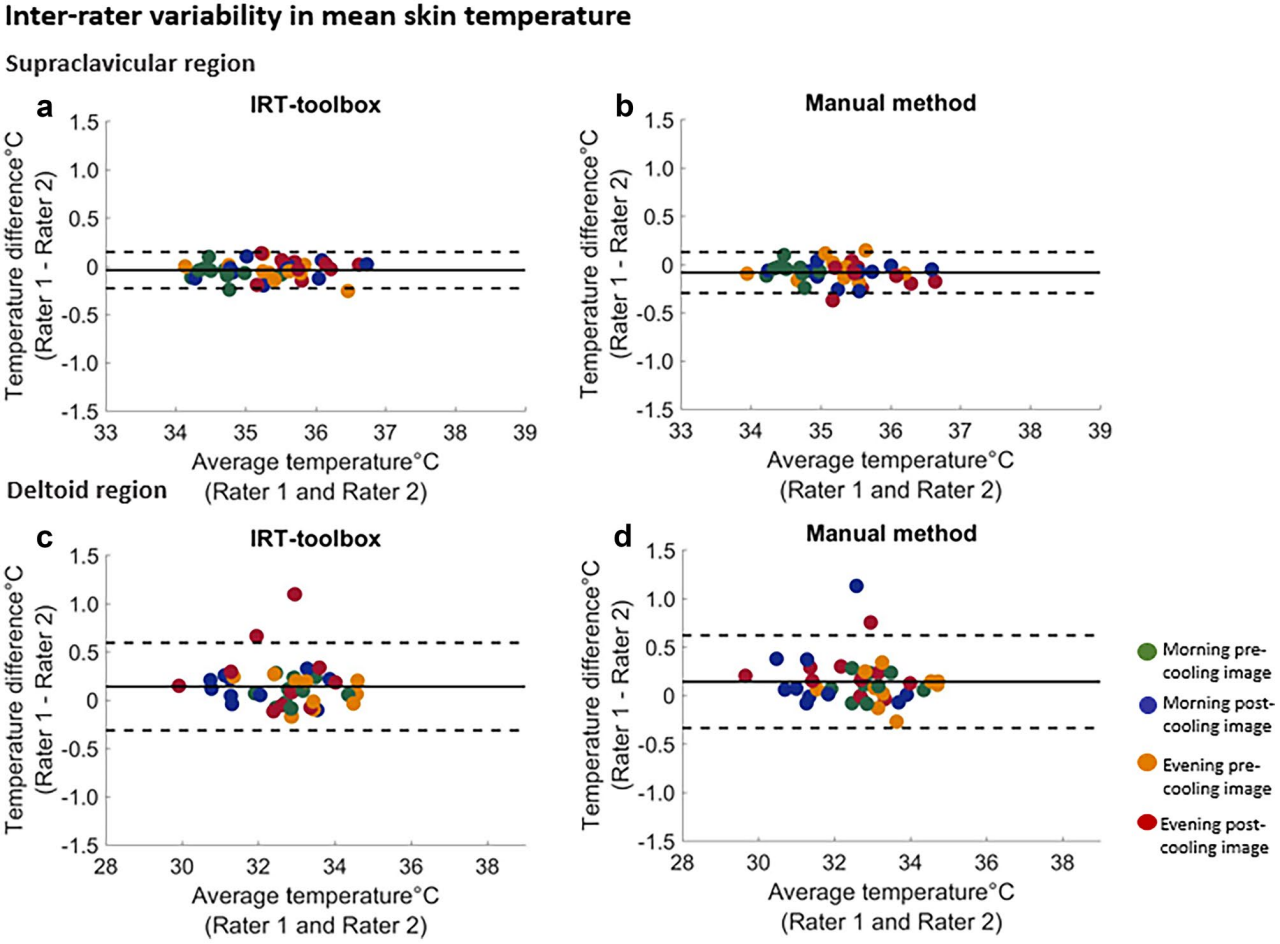
The mean difference and variability in mean skin temperature between raters The mean difference and variability in mean skin temperature in the supraclavicular area **a, b** and deltoid area **c, d** between two raters using the IRT-toolbox and the manual method are shown. Data for all ten participants are shown; different colors indicate different imaging sessions. The solid line represents the mean difference and the dashed lines represent the upper and the lower 95% limits of agreement

Regarding the skin temperature outcomes between the two raters, the mean supraclavicular skin temperature differences between raters were -0.04 °C, 95% LoA = [-0.23 °C,0.14 °C] using the IRT-toolbox, and -0.09 °C, 95% LoA = [-0.30 °C, 0.12 °C] using manual segmentations; see Fig. 3a, b.

The mean deltoid skin temperature difference between raters was 0.14 °C, 95% LoA = [-0.32 °C, Dice coefficient is presented as mean, 0.59 °C] using the IRT-toolbox, and 0.14 °C, 95% LoA = [-0.33 °C, 0.62 °C]) with manual segmentations; see Fig. 3c, d. The results for the maximum skin temperature are shown in Online resource 1; Fig. S2. Temperature gradients in the supraclavicular area and the deltoid region were determined to evaluate the homogeneity of the temperature distributions in both areas. Results are shown in Online resource 1; Fig. S3.

## Discussion

In this work, we developed an open-access semi-automated segmentation method, and compared it to manual delineations. The IRT-toolbox effectively reduced the ROI drawing time to a single image per dataset. Importantly, our method showed a similar inter-user variability to manual segmentations.

In previous work, semi-automated ROI delineations took longer when moderate displacements were present [11]: in a semi-automated analysis without non-rigid registration, manual input is still needed on all images. Our results show that the image pre-alignment and image registration steps of the IRT-toolbox successfully accounted for displacements. This enabled ROI drawing on the reference image only, and therefore the total drawing time was reduced.

The mean spatial overlap between the reference ROI and manually delineated ROIs was good for the supraclavicular region, and moderate for the deltoid region. For both regions, however, a large variability in spatial overlap was found between methods, which is likely due to differences in ROI placement and size of manually drawn follow-up ROIs. The IRT-toolbox minimizes such variability by utilizing a single ROI applied to all registered follow-up images. Although a large variability was found in ROI placement between methods, skin temperature differences were relatively small (< 1 °C) for both areas.

The IRT-toolbox showed the same inter-user variability in skin temperature outcomes as manual segmentations in both areas. The IRT-toolbox improved the spatial agreement between the ROIs from the two raters in the deltoid area compared to manual segmentations (good versus moderate), whereas both methods scored the same for the supraclavicular area (good). This difference may be due to the larger size of the deltoid area compared to the supraclavicular area.

The variability in ROI placement and ROI size between methods and raters do not seem to influence differences in skin temperature outcomes, most likely due to relatively small temperature gradients between the region of interest and surrounding tissues (Online resource 1; Fig. S3). Hence, the IRT-toolbox may further minimize the variability between users in future applications that involve tissues with more heterogenous temperature distributions.

### Practical Implications, Limitations and Future Directions

The IRT-toolbox is an open-access, freely available method for temperature analyses and available for clinical applications. The semi-automated part of the program reduces drawing time to a single image per participant, which makes it favorable in studies with repeated measurements. A limitation of this study is that no calibration procedures were performed prior to imaging, and no corrections were made for the environmental variance between imaging conditions. This will not have influenced our results since we did not determine skin temperature differences between different imaging conditions (i.e., morning pre-cooling and evening pre-cooling), but determined skin temperature differences between segmentation methods and raters where ROIs were applied to the same image for comparison. Nevertheless, we do recommend to perform these kind of corrections to enable more accurate estimates of supraclavicular skin temperature changes between different imaging sessions. In addition, the detected pixel in the IRT image had a size of 0.2 cm based on the instantaneous field of view (iFOV) of 1.308 mrad and a target distance of 1.5 meters [17, 19]. The thermal measurement area that corresponded to 1 cm^2^ consisted of 25 pixels. It should be taken into account that this measurement area had a minimal variation in the two-dimensional IRT image since the body surface is not flat and the pixel area is not infinitely small. A limitation of the IRT-toolbox is the registration time. However, this step has to be performed only once in the analysis and a built-in function of the program allows users to automatically run the image registration part consecutively on multiple datasets. Another limitation is that the IRT-toolbox can only be used in thermal images, where anatomical regions have a sufficiently different temperature compared to the background, such as in imaging the feet in diabetes [20]. In this case, an additional color (*i.e.,* Red Green Blue, RGB) image might need to be integrated. The IRT-toolbox may be optimized by automating the method using e.g., skin fiducials, anatomical landmarks and/or artificial networks, and combined with high-end computers. This will further minimize user workload and may fully eliminate the variability between raters.

In conclusion, we introduced a new semi-automated segmentation tool to facilitate temperature analyses of supraclavicular and deltoid skin temperatures. The IRT-toolbox reduced the ROI delineation time and showed a comparable inter-user variability with respect to manual segmentations.

## Supplementary Information

Below is the link to the electronic supplementary material.

Supplementary file1 (MP4 133670 KB)

Supplementary file2 (PDF 585 KB)

## Data Availability

Data is available from the corresponding author on reasonable request. The IRT-toolbox and a video tutorial will be made freely available for download at https://github.com/AashleySD/IRT_toolbox/ and Online resource 2.

## Abbreviations

BAT: Brown adipose tissue
BMI: Body mass index
IRT: Infrared thermography
LoA: Limits of agreement
RGB: Red Green Blue
ROI: Region of interest
