# Supplementary material for: The Infrared Thermography Toolbox: An Open-access Semi-automated Segmentation Tool for Extracting Skin Temperatures in the Thoracic Region including Supraclavicular Brown Adipose Tissue
Source: J Med Syst. 2022 Nov 2;46(12):89. doi: 10.1007/s10916-022-01871-7 (PMC9626432; doi:10.1007/s10916-022-01871-7)
Supplement: Supplementary file 2 — Supplementary file2 (PDF 585 KB) [file 10916_2022_1871_MOESM2_ESM.pdf]

Supplemental figures

The infrared thermography toolbox: an open-access semi-automated segmentation tool for extracting thoracic skin temperatures

Journal of medical systems

Aashley S.D. Sardjoe Mishre[1,2], Maaike E. Straat[1], Borja Martinez-Tellez[1,5], Andrea Mendez Gutierrez[6,7], Sander Kooijman[1], Mariëtte R. Boon[1], Oleh. Dzyubachyk[3,4], Andrew Webb[2], Patrick C. N. Rensen[1], Hermien E. Kan[2]

Affiliations:

1. Department of Medicine, Division of Endocrinology, Leiden University Medical Center, Leiden, the Netherlands.
2. Department of Radiology, C.J. Gorter Center for High Field MRI, Leiden, the Netherlands.
3. Department of Radiology, Division of Image Processing (LKEB), Leiden University Medical Center, Leiden, The Netherlands
4. Department of Cell and Chemical Biology, Section Electron Microscopy, Leiden University Medical Center, Leiden, The Netherlands
5. Department of Education, Faculty of Education Sciences and SPORT Research Group (CTS-1024), CERNEP Research Center, University of Almería, Almería, Spain
6. Department of Biochemistry and Molecular Biology II, "José Mataix Verdú" Institute of Nutrition and Food Technology (INYTA), Biomedical Research Centre (CIBM), University of Granada, Granada, 18016, Spain.
7. Biohealth Research Institute in Granada (ibs. GRANADA), Granada, 18012, Spain. CIBER Fisiopatología de la Obesidad y la Nutrición (CIBEROBN), Madrid, 28029, Spain

Corresponding author:
Dr. Hermien E. Kan
Email address: H.E.Kan@lumc.nl

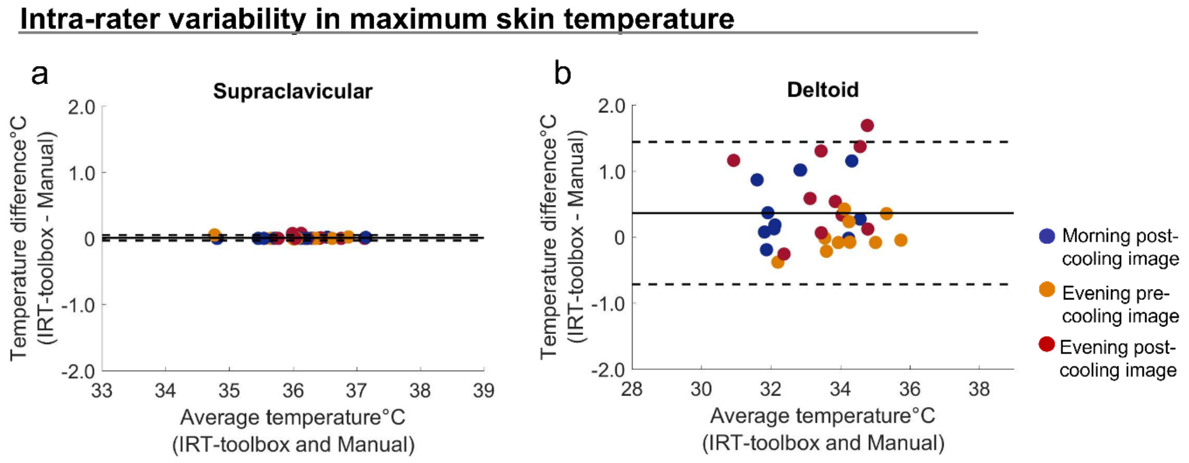

Supplemental Figure 1. The mean difference and variability in maximum skin temperature between methods

The mean difference and variability in maximum skin temperature between methods for the supraclavicular area (**a**) and the deltoid area (**b**) are shown. Data for all ten participants are shown; different colors represent different imaging conditions. The solid line represents the mean difference and the dashed lines represent the upper and the lower 95% limits of agreement.

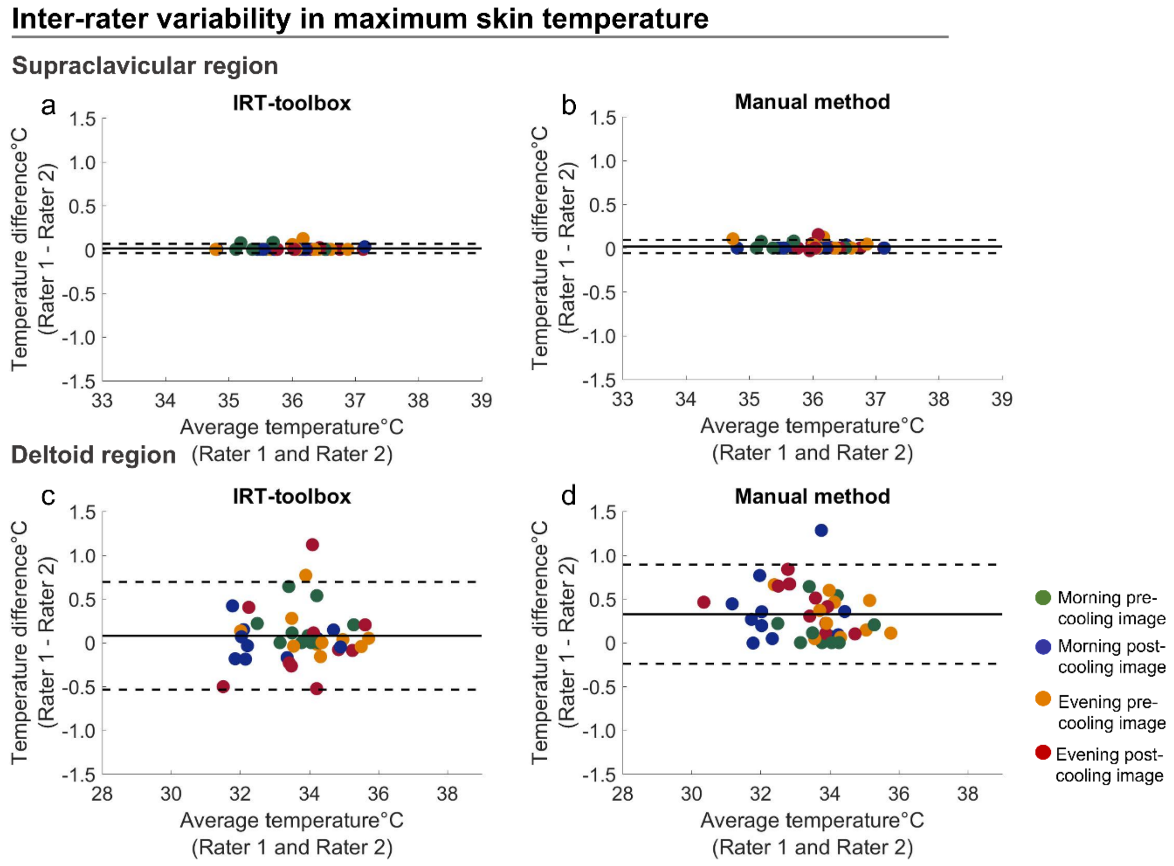

Supplemental Figure 2. The mean difference and variability in maximum skin temperature between raters

The mean difference and variability in maximum skin temperature in the supraclavicular area (**a**, **b**) and deltoid area (**c**, **d**) between two raters using the IRT-toolbox and the manual method is shown. Data for all ten participants are shown; different colors indicate different imaging conditions. The solid line represents the mean difference and the dashed lines represent the upper and the lower 95% limits of agreement.

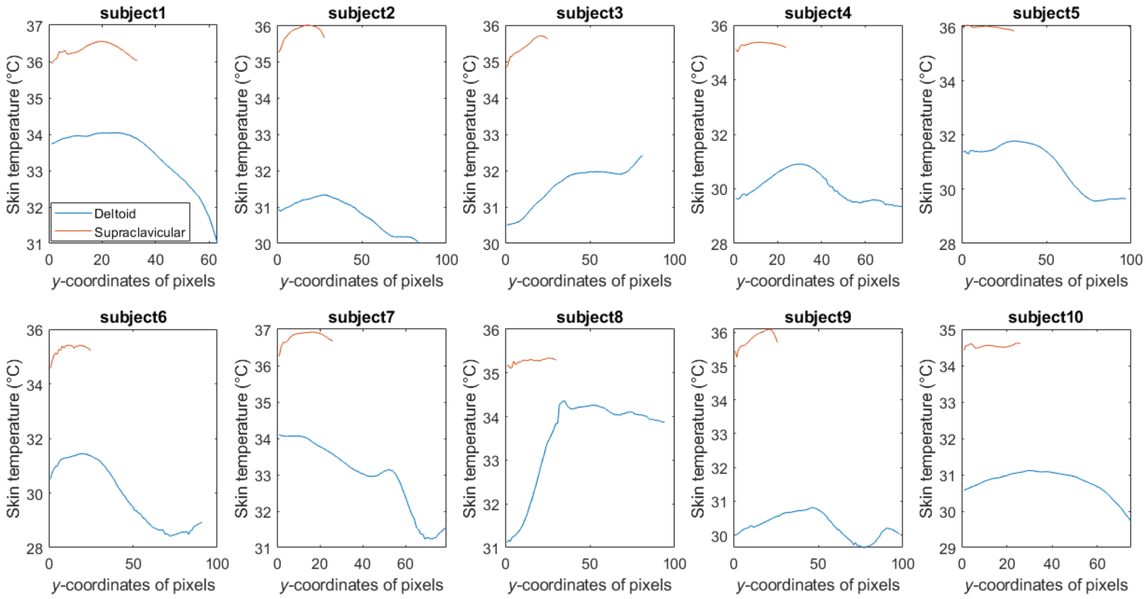

Supplemental Figure 3. Temperature distribution in the deltoid and supraclavicular regions.

Skin temperature values along a vertical line passing through the deltoid region (blue) and the supraclavicular region (red) are plotted.
